# Warming reduces tall fescue abundance but stimulates toxic alkaloid concentrations in transition zone pastures of the U.S.

**DOI:** 10.3389/fchem.2014.00088

**Published:** 2014-10-21

**Authors:** Rebecca L. McCulley, Lowell P. Bush, Anna E. Carlisle, Huihua Ji, Jim A. Nelson

**Affiliations:** Department of Plant and Soil Sciences, University of KentuckyLexington, KY, USA

**Keywords:** climate change, *Epichloë coenophiala*, ergot alkaloids, loline alkaloids, *Lolium arundinaceum*, pasture sustainability

## Abstract

Tall fescue pastures cover extensive acreage in the eastern half of the United States and contribute to important ecosystem services, including the provisioning of forage for grazing livestock. Yet little is known concerning how these pastures will respond to climate change. Tall fescue's ability to persist and provide forage under a warmer and wetter environment, as is predicted for much of this region as a result of climate change, will likely depend on a symbiotic relationship the plant can form with the fungal endophyte, *Epichloë coenophiala*. While this symbiosis can confer environmental stress tolerance to the plant, the endophyte also produces alkaloids toxic to insects (e.g., lolines) and mammals (ergots; which can cause “fescue toxicosis” in grazing animals). The negative animal health and economic consequences of fescue toxicosis make understanding the response of the tall fescue symbiosis to climate change critical for the region. We experimentally increased temperature (+3°C) and growing season precipitation (+30% of the long-term mean) from 2009–2013 in a mixed species pasture, that included a tall fescue population that was 40% endophyte-infected. Warming reduced the relative abundance of tall fescue within the plant community, and additional precipitation did not ameliorate this effect. Warming did not alter the incidence of endophyte infection within the tall fescue population; however, warming significantly increased concentrations of ergot alkaloids (by 30–40%) in fall-harvested endophyte-infected individuals. Warming alone did not affect loline alkaloid concentrations, but when combined with additional precipitation, levels increased in fall-harvested material. Although future warming may reduce the dominance of tall fescue in eastern U.S. pastures and have limited effect on the incidence of endophyte infection, persisting endophyte-infected tall fescue will have higher concentrations of toxic alkaloids which may exacerbate fescue toxicosis.

## Introduction

Tall fescue [*Lolium arundinaceum* (Schreb.) Darbysh, a.k.a. *Festuca arundinacea* (Schreb.), and *Schedonorus arundinaceus* (Schreb.) Dumort.] is a C_3_ physiology, cool-season perennial grass that was introduced to North America from Eurasia in the late 1800s, but today covers more than 14 million hectares, primarily in the eastern half of the United States (Ball et al., 1993; Hoveland, 2009; Young et al., 2013). In this region, tall fescue is widely utilized as a forage in pasture-based livestock systems, in part due to its ease of establishment, environmental hardiness, and ability to persist and produce forage under a range of management regimes (Roberts et al., 2009). Many of these attributes are linked to a symbiotic relationship the plant can form with an asymptomatic, asexual fungal endophyte—*Epichloë coenophiala* (a.k.a., *Neotyphodium coenophialum*; Clay, 1990; Schardl et al., 2004; Leuchtmann et al., 2014).

Similar grass—fungal endophyte relationships are common (Arnold et al., 2000), occurring on every continent except Antarctica and within 20–30% of grass species (White, 1987; Leuchtmann, 1992). The nature of these relationships varies across the symbiotic continuum (Saikkonen et al., 1998), depending on plant and fungal genetics and biotic and abiotic environmental parameters (Cheplick and Faeth, 2009). While the relationship between tall fescue and *E. coenophiala* is generally considered mutualistic (Clay, 1990), prior work suggests that the benefits of fungal endophyte infection to the plant might be most pronounced under conditions of high herbivory (Bouton et al., 1993; Clay et al., 2005), high or low nutrient availability (Malinowski and Belesky, 2000; Rahman and Saiga, 2005), and/or at times of extreme heat or water limitation, such as commonly occurs during summer droughts (Arachevaleta et al., 1989; Elbersen and West, 1996; Marks and Clay, 1996; Assuero et al., 2006).

Tall fescue is thought to provide food, shelter, and a means of reproduction for the fungus, as *E. coenophiala* can only infect new tall fescue individuals through vertical, mother-daughter transmission (Clay and Schardl, 2002; Schardl et al., 2004). While in return, the fungus produces a suite of alkaloids, including peramines, lolines, and ergots—some of which are known to deter herbivory (Bush et al., 1997). Because these fungal-produced alkaloids are toxic to some herbivores, their presence can alter herbivory [e.g., cause increased consumption of surrounding endophyte-free (E−) individuals or other plant species] such that a significant competitive advantage is conferred to endophyte-infected (E+) tall fescue (Clay and Holah, 1999; Clay et al., 2005). Unfortunately, grazing livestock are susceptible to these alkaloids, and significant negative animal health issues arise when animals are forced to graze infected tall fescue material, particularly during times of temperature stress (i.e., fescue toxicosis in the southeastern U.S.; Stuedemann and Hoveland, 1988; Hoveland, 1993; Strickland et al., 2011).

However, the tall fescue—*E. coenophiala* symbiosis is not just a defensive mutualism (Clay and Schardl, 2002). While studies have shown that in situations where herbivory levels are high, the percentage of fungal endophyte infected individuals within a tall fescue community can increase over time (Clay et al., 2005), fungal endophyte infection frequencies (EIF) have also been shown to vary in relation to environmental stress (Lewis et al., 1997). Higher EIF are frequently encountered in harsher environments (West et al., 1993), and for tall fescue, survival, and recovery following exposure to high temperatures and water limitation are often enhanced by endophyte infection (Elmi and West, 1995; Elbersen and West, 1996; Marks and Clay, 1996; Belesky and West, 2009). Such results suggest that endophyte infection may improve tall fescue's ability to resist and adapt to environmental perturbations that are likely to result from climate change—a supposition made for many symbiotic associations (Compant et al., 2010; Redman et al., 2011; Kivlin et al., 2013).

Only a few studies to date have experimentally evaluated this possibility for tall fescue and results are mixed. Endophyte infection sometimes alters tall fescue response to elevated atmospheric CO_2_ (Newman et al., 2003) but not always (Chen et al., 2007). Effects of endophyte infection on plant recovery and survival following drought and temperature stress, while somewhat variable, tend to be positive for tall fescue (Arachevaleta et al., 1989; Marks and Clay, 1996; Worchel et al., 2013). Elevated CO_2_ has been shown to reduce, while drought and increased temperature stimulate, alkaloid concentrations in E+ material (Agee and Hill, 1994; Brosi et al., 2011b; Ryan et al., 2014). Long-term field manipulations of multiple climate change factors are rare, and yet they are essential to understanding how complex systems will respond (Backlund et al., 2008). In one such study, Brosi et al. (2011b) found that elevated CO_2_ led to a higher EIF within the tall fescue community of a mixed species, unmanaged old field after 5 years of experimental manipulation, but surprisingly, measured no change in EIF under drought or elevated temperature treatments or their interactions. Unfortunately, this study could not capture changes in tall fescue—endophyte dynamics over time and was not representative of pasture conditions (species- or management-wise); therefore, implications from it for tall fescue pastures are limited.

Given that tall fescue is a dominant component of pastures across extensive acreage in the eastern U.S., and serves as the forage base for a multi-million dollar animal industry, which suffers substantially from the negative effects of fungal-produced alkaloids, it is important to understand how potential changes in climate will impact the fescue—endophyte symbiosis. This project evaluated the consequences of warming (+3°C, day and night, year-round) and increased growing season precipitation (+30% of the long-term mean), conditions that are representative of current climate projections for this region (Brunjes, 2004; Karl et al., 2009), on tall fescue abundance, EIF, and alkaloid concentrations over a 5 years period in a hay-managed, mixed species pasture in central Kentucky. Because tall fescue is a cool season grass and the warming treatment was expected to increase the intensity and duration of seasonal dry periods normally experienced at the site, we anticipated that warming would decrease tall fescue abundance, especially in mid-summer and early fall, and that this environmental stress would select for E+ individuals and thereby increase the level of endophyte infection within the tall fescue community over time. We also hypothesized that warming would stimulate alkaloid concentrations in E+ individuals and that the addition of precipitation would ameliorate some of these effects of warming on the plant and its symbiotic partner.

## Materials and methods

### Site description

The climate change project was established in an existing pasture, on relatively deep (>1 m) Maury silt loam soils, located at the University of Kentucky's Spindletop Farm, Lexington, KY, USA (38.1081°N; 84.4916°W). The site is situated in a transition zone between subtropical and continental climates, experiencing long-term mean annual summer and winter temperatures of 23.8 and 1.6°C, respectively (Ferreira et al., 2010), with rainfall relatively evenly distributed throughout the year, averaging 1137 mm a year. Average annual temperatures (°C) and total annual rainfall (mm) were as follows, respectively, over the 5 years project: 2009—12.7, 1323; 2010—13.2, 1048; 2011—13.4, 1656; 2012—14.4, 1019; and 2013—12.8, 1495 (Kentucky Mesonet—Lexington).

In March and April 2008, the pasture was sprayed with glyphosate, plowed, and disked to remove existing vegetation and to prepare a seedbed. A seed mixture consisting of cool season, C_3_ physiology forage species common to the area were planted on April 8, 2008 and consisted of: Kentucky bluegrass (*Poa pratensis*, cultivar “Ginger”), tall fescue (cultivar “Kentucky-31”), red clover (*Trifolium pretense*, cultivar “Freedom”), and white clover (*Trifolium repens*, cultivar “Patriot”). For tall fescue, 50% of the seed was infected with the common toxic strain of *E. coenophialum* and 50% was not infected (i.e., was *Epichloë*-free). On August 22, 2008, the warm-season, C_4_ physiology forage grass, Bermuda (*Cynodon dactylon*, cultivar “Wrangler”), was plugged throughout the establishing stand. Plugs originated from an existing adjacent field. For more details on site characteristics and stand establishment see Brosi (2011a), Slaughter (2012), and Bourguignon (2013).

### Experimental design

Within the newly established stand (~1500 m^2^), five replicate blocks, similar in vegetative composition and large enough to contain four, 3 m diameter (5.8 m^2^) plots, were identified. Plots within a block were randomly assigned to one of four climate treatments: +heat (+3°C above ambient temperature); +precip (+30% long-term normal precipitation; +343 mm); +heat+precip (the combination of the first two treatments); and an untreated, ambient control. The +heat treatment was achieved by following the approach of Kimball et al. (2008). Salamander infrared ceramic heaters (Mor Electric Heating Assoc., Comstock Park, MI) were positioned around each plot, and heat was applied as needed to maintain the desired +3°C difference between the +heat plot and its paired ambient temperature comparison (control plots for +heat treatment and +precip plots for the +heat+precip treatment). To control for potential shade effects from the heaters, all plots were surrounded by the housing units used to mount them. For the +precip treatment, rainfall collected on-site was applied using a metered wand, twice a month, on rainy days, during the growing season only (April to September). Quantities of additional precipitation applied each month were based on long-term rainfall trends for the site and were constant across years of the experiment (April +50.8 mm; May +50.8 mm; June +61.7 mm; July +67.0 mm; August +61.7 mm; September +50.8 mm). Aluminum sheeting was inserted into the soil to 50 cm depth around individual plots to minimize movement of soil moisture into or out of the plots. Climate treatments began May 1, 2009 and ran continuously until November 12, 2013.

### Measurements

Effects of the climate treatments on air temperature and soil moisture were measured continuously throughout the experiment. Air temperature was measured every 15 s at 30 cm above the ground surface with shielded Type T thermocouples (FW3648, TE Wire, Saddle Brook, NJ). Volumetric water content of the top 15 cm of soil was measured every 15 min using time domain reflectrometers (CS-616, Campbell Scientific, Logan, UT). Daily, monthly, and seasonal averages of these data for each treatment were computed.

The experiment was managed as a hay field, meaning that all vegetation was mowed to a height of 7.6 cm, and cut material was removed off site. For all years of the project, mowing occurred three times per growing season. Climate treatment effects on total aboveground plant biomass production (data not shown) and the biomass and relative abundance of tall fescue were obtained from seasonal sub-plot harvests that immediately preceded whole-plot mowing events. All vegetation located within two, 0.25 m^2^ permanent sub-plots per plot was cut to 7.6 cm height, removed, and sorted by hand to species. This material was then oven-dried (55°C for 3–4 days) and weighed. The relative abundance of tall fescue was calculated on a biomass basis: tall fescue biomass/total biomass per sub-plot. Sub-plot values were averaged for a plot value for subsequent statistical analyses. Harvests occurred on the following dates: 2009 (June 1, July 21, Sept. 22); 2010 (May 17, July 26, Oct. 5); 2011 (May 23, July 25, Oct. 5); 2012 (May 21, July 30, Oct. 8); and 2013 (May 21, Aug. 5, Oct. 14).

In order to determine whether the climate treatments were affecting the occurrence of E+ and E− individuals within the tall fescue population, ~40 tall fescue tillers per plot were harvested by hand, at 7.6 cm above ground level, with a razor blade, immediately preceding all spring and fall harvests. These tillers were kept cold in a freezer (−4.0°C) until they could be processed for endophyte presence (usually within 1–2 weeks). We utilized an enzyme-linked, endophyte-specific immunosorbent assay to determine whether individual tillers were E+ or E− (Hiatt et al., 1999). Each tiller was individually labeled and double-blotted onto nitrocellulose paper that was then assayed for the presence of the endophyte-specific immunoprotein. If the endophyte protein was present, a color reaction would occur, and tiller blots would turn pink. They remained colorless if no endophyte protein was detected. Plot-level tall fescue EIF was calculated as the number of tillers testing positive for the presence of the endophyte divided by the total number of tillers tested per plot. One person scored all the blots as either positive or negative for the entire project, and positive and negative controls were run with each batch. Because, like others (Ju et al., 2006), we detected regular seasonal patterns in EIF (spring values usually lower than fall values, across all treatments; data not shown), we present only the initial June 2009 and all following fall EIF data. Our treatment-related EIF results do not change if all data are included in the statistical models. Fescue tillers were lyophilized once the blots were scored.

Because only the fungal endophyte can produce the insect and mammal toxic alkaloids we were interested in measuring (Bush et al., 1997), the immunoblot results were used to separate the E+ tillers from the E− before alkaloid analysis. Only E+ tillers were composited per plot, ground to pass through a 1 mm screen using a Cyclotec 1093 mill, and analyzed for loline and ergot alkaloid concentrations. Due to lab error, we did not measure alkaloids on the first fescue tiller harvest (June 2009), but they were measured on all E+ tillers collected thereafter. Gas chromatography (GC) was used to identify and quantify three loline compounds (*N*-formyl loline, *N*-acetyl loline, and *N*-acetyl norloline), according to the protocol of Blankenship et al. (2001). A 0.3 g sub-sample of ground material was extracted in sodium bicarbonate and methylene chloride containing quinoline (15 μg mL^−1^; an internal standard) via shaking for an hour. Extracts were filtered and analyzed on a GC (Perkin Elmer Clarus 500) equipped with a flame ionized detector and using an SPB-1 fused silica capillary column (15 m × 0.53 mm, 0.5 μm film thickness; Supelco). The GC temperature program increased from 80 to 160°C at 20°C min^−1^, was held for 2 min, was increased to 290°C at 45°C min^−1^, and held for 5 min. Injector and detector temperatures were set at 250 and 275°C, respectively.

High performance liquid chromatography (HPLC) with florescence detection was employed to quantify ergovaline and ergovalinine concentrations, as developed by Yates and Powell (1988). A 0.1 g ground sample was extracted in 80% methanol via shaking for 2 h. The extractant was then forced, by syringe, through a PreSep column (SPE, C18 disposable) fitted with a 0.2 μm polytetrafluoroethylene filter. The third mL aliquot of extractant was isolated and eluted with the following solutions: (A) 0.1 M ammonium acetate: acetonitrile, 97:3 v/v and (B) 100% acetonitrile. A reverse phase Kinetex XB-C18 column (100 × 4.6 mm with 2.6 μm particle size) was used to separate compounds at a flow of 1.2 mL min^−1^ with the following gradient conditions: initial 22% mobile phase B increased linearly to 35% over 20 min and then further linearly to 58% B in 8 min before being increased to 100% B and held for 5 min. After this period, re-equilibration was achieved by reducing to 22% B for 9 min. Ergovaline and ergovalinine were identified by excitation at 310 and emission measurement at 420 nm and had retention times of 14.1 and 24.4 min, respectively. Endophyte-free samples were included as checks periodically and consistently had non-detectable levels of all the compounds of interest. Independently verified E+ material, not associated with this project, was run during all analyses as a lab standard.

### Statistical analysis

The randomized complete block design of the project included five replicates of four climate change treatments that were implemented in a 2 × 2 factorial design (+heat × +precip), with measurements taken over a 5-years period of time. Therefore, we statistically analyzed majority of our data with a repeated measures analysis of variance mixed linear model using the restricted maximum likelihood method within Proc Mixed procedures of SAS version 9.3 (SAS Institute Inc., Cary, NC). Main, fixed effects were designated as heat, precip, time, and all interactions therein. Block was the random effect, and the repeated subject was block by climate treatment. All parameters were tested for normality and homogeneity of variance and adjusted, if necessary, as follows.

The time variable, and therefore the repeated measure, differed across parameters. For air temperature and soil moisture, average seasonal values were calculated for each year (Spring—March, April, May; Summer—June, July, August; Fall—September, October, November; Winter—December, January, February). Because we knew years varied significantly in these parameters, we analyzed this seasonal data by year (2009–2013) using a multiple analysis of variance model (Proc GLM; SAS v.9.3), with block, heat, and precipitation as main effects and season as the repeated variable. For tall fescue relative abundance, harvest period (Spring, Summer, and Fall) and year were included as the time variables in the models. For alkaloid concentrations, material sampled was from two seasons (Spring and Fall) over the 5 years, so both season and year were included in the models.

To determine whether changes in EIF occurred over time in the climate treatments, we used time as a continuous measure (months since experiment began), and calculated the difference in EIF between the month of measure and the initial infection frequency on a per plot basis, which was then analyzed with the same model as the other parameters. To assess whether differences in EIF existed at the start of the experiment, the June 2009 EIF data were angular transformed, to meet assumptions of the model, and run with heat, precip, and their interaction as main effects, block as the random effect, and no repeated statement. There were no significant differences in EIF across plots at the initial measurement period (all *p* > 0.4), with the site averaging 41.1% (±3.3) of fescue tillers infected.

For significant main effects and interactions (*p* < 0.05), mean comparisons were performed with lsmeans using either the pdiff or the Dunnett multiple comparison adjustment (for EIF data only). Because we did not measure alkaloids the first spring (2009), we ran means comparison tests on these data with year 2009 excluded. For air temperature and soil moisture data, Type I sums of squares were used to generate p-values for the climate treatment effects (heat, precip, and their interaction), and for season and its interactions with the climate treatments, Hotelling-Lawley Trace method was used to generate the *p*-values.

## Results

### Air temperature and soil moisture

**Figure 1:**
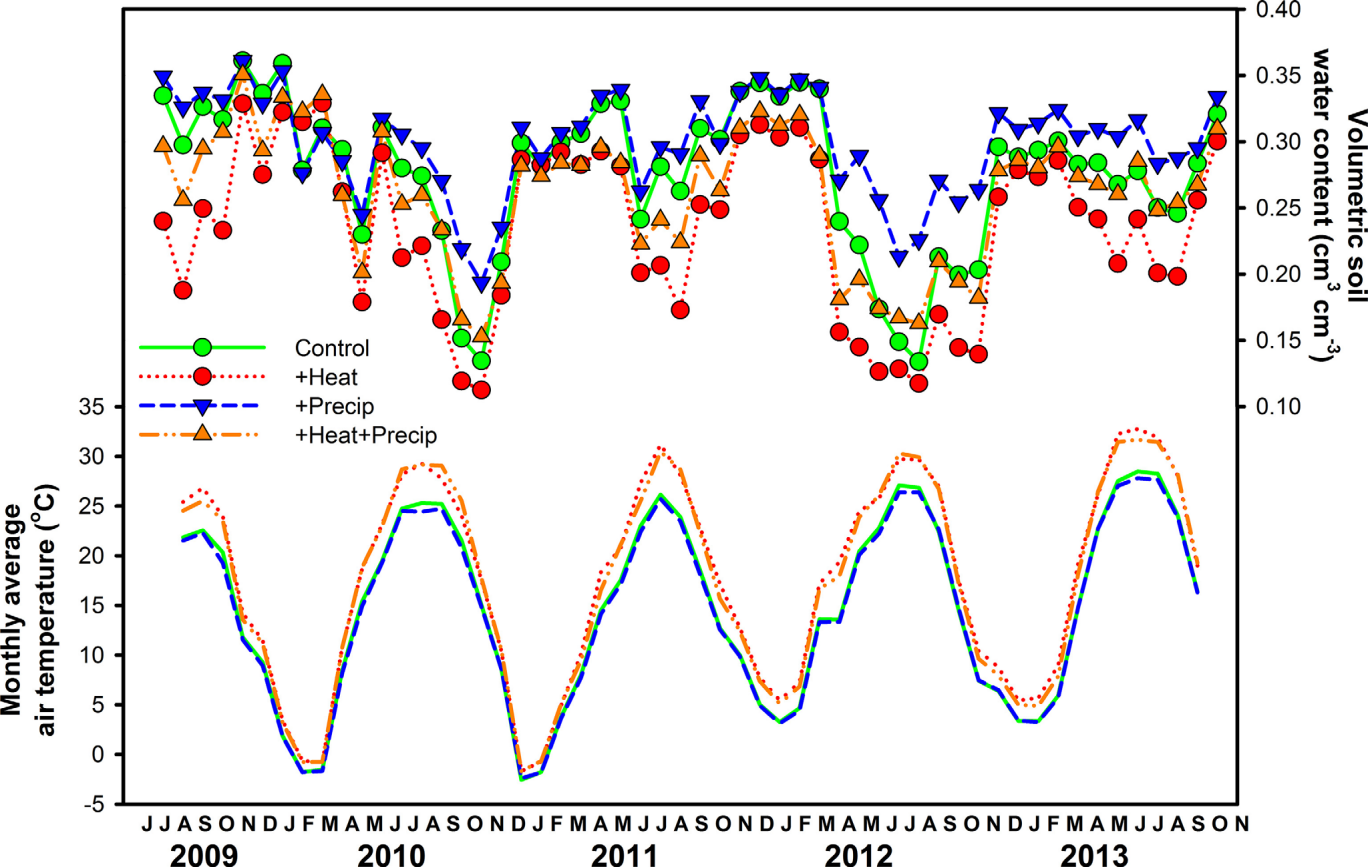
**Average (±SE) monthly air temperature (lines only) and volumetric soil moisture (0–15 cm; lines plus symbols) measured in each plot of the four climate treatments (ambient control, +heat, +precip, +heat+precip) across the 5 years study period**.

As expected, given the intention of the climate manipulations, air temperatures were increased by 3.04 ± 0.17°C in +heat plots vs. the ambient temperature treatments, averaged (±SE) across the entire 5-years project (Figure 1). Increases in air temperature due to warming were greater during the summer (3.87 ± 0.22°C) than the winter (1.63 ± 0.13°C), explaining the significant season × heat effect in all years of the project (Table 1), but the magnitude of this seasonal effect varied across years (Figure 1).

**Table 1:** **Statistical results of the effects of season, the climate treatments (+heat and +precip), and their interactions on air temperature and soil moisture across the 5-years project period, 2009–2013**.

**Effect**	**2009**			**2010**			**2011**			**2012**			**2013**		
	***df***	***F*-value**	**Pr > *F***	***df***	***F*-value**	**Pr > *F***	***df***	***F*-value**	**Pr > *F***	***df***	***F*-value**	**Pr > *F***	***df***	***F*-value**	**Pr > *F***
**AIR TEMPERATURE**															
Heat	1	102.92	**<0.0001**	1	195.18	**<0.0001**	1	260.61	**<0.0001**	1	160.31	**<0.0001**	1	87.20	**<0.0001**
Prec	1	3.04	0.1069	1	0.00	0.9839	1	5.13	**0.0429**	1	1.41	0.2577	1	0.70	0.4180
Heat^*^Prec	1	0.23	0.6386	1	2.30	0.1556	1	0.62	0.4447	1	0.20	0.6638	1	0.03	0.8603
Seas	2,11	10848.5	**<0.0001**	3,10	15135.3	**<0.0001**	3,10	11160.9	**<0.0001**	3,10	14452.7	**<0.0001**	2,11	5012.8	**<0.0001**
Seas^*^Heat	2,11	31.75	**<0.0001**	3,10	37.98	**<0.0001**	3,10	31.96	**<0.0001**	3,10	22.92	**<0.0001**	2,11	4.95	**0.0294**
Seas^*^Prec	2,11	2.12	0.1666	3,10	0.51	0.6815	3,10	1.86	0.2005	3,10	1.97	0.1826	2,11	2.65	0.1150
Seas^*^Heat^*^Prec	2,11	0.55	0.5918	3,10	3.25	0.0682	3,10	0.71	0.5684	3,10	5.49	**0.0172**	2,11	0.22	0.8040
**SOIL MOISTURE**															
Heat	1	33.07	**<0.0001**	1	14.82	**0.0023**	1	11.26	**0.0057**	1	17.06	**0.0014**	1	7.77	**0.0164**
Prec	1	11.03	**0.0061**	1	6.60	**0.0246**	1	1.03	0.3302	1	9.85	**0.0086**	1	5.79	**0.0331**
Heat^*^Prec	1	5.80	**0.0330**	1	0.01	0.9323	1	0.09	0.7749	1	0.44	0.5215	1	0.04	0.8459
Seas	2,11	65.76	**<0.0001**	3,10	1218.62	**<0.0001**	3,10	357.86	**<0.0001**	3,10	622.09	**<0.0001**	2,11	103.96	**<0.0001**
Seas^*^Heat	2,11	76.26	**<0.0001**	3,10	9.40	**0.0029**	3,10	11.67	**0.0013**	3,10	44.67	**<0.0001**	2,11	7.19	**0.0101**
Seas^*^Prec	2,11	11.76	**0.0019**	3,10	37.58	**<0.0001**	3,10	7.66	**0.0060**	3,10	22.32	**<0.0001**	2,11	22.74	**0.0001**
Seas^*^Heat^*^Prec	2,11	2.78	0.1053	3,10	9.98	**0.0024**	3,10	0.85	0.4996	3,10	3.36	0.0633	2,11	3.10	0.0855

*Significant p-values are bolded*.

Warming reduced soil moisture by 13.9% averaged over the duration of the project, but this effect was strongest during the summer period, especially in the drier years—2010 and 2012 (Figure 1, Table 1). The +precip treatment mediated, to some degree, the effects of warming during the summer dry-down period: +heat+precip plots averaged 24.3% more soil moisture during the summer than +heat plots. However, +heat+precip plots rarely had greater soil moisture then ambient control plots (Figure 1). Plots receiving only the +precip treatment were the wettest, especially during the summer, when the additional water was being applied (averaging 15.2–50.7% more soil moisture than ambient controls or +heat treatments in all summers of the project). Soils were generally wettest in the winter and driest during the summer, though considerable year-to-year variability in soil moisture was encountered [average annual (or less than annual in some years) soil moisture across treatments ± SE: 31.1 ± 0.12%—2009 (June–Dec.); 24.8 ± 0.14%—2010; 28.7 ± 0.27%—2011; 23.3 ± 0.30%—2012; and 27.6 ± 0.14%—2013 (Jan.–Oct.)]. In 2011, the wettest year of the project, additional precipitation had no effect on soil moisture, even during the summer (although the season × precip interaction was significant, means comparison tests failed to identify a significant comparison), but slightly cooled the plots receiving this treatment (Table 1).

### Tall fescue relative abundance and endophyte infection frequency

**Figure 2:**
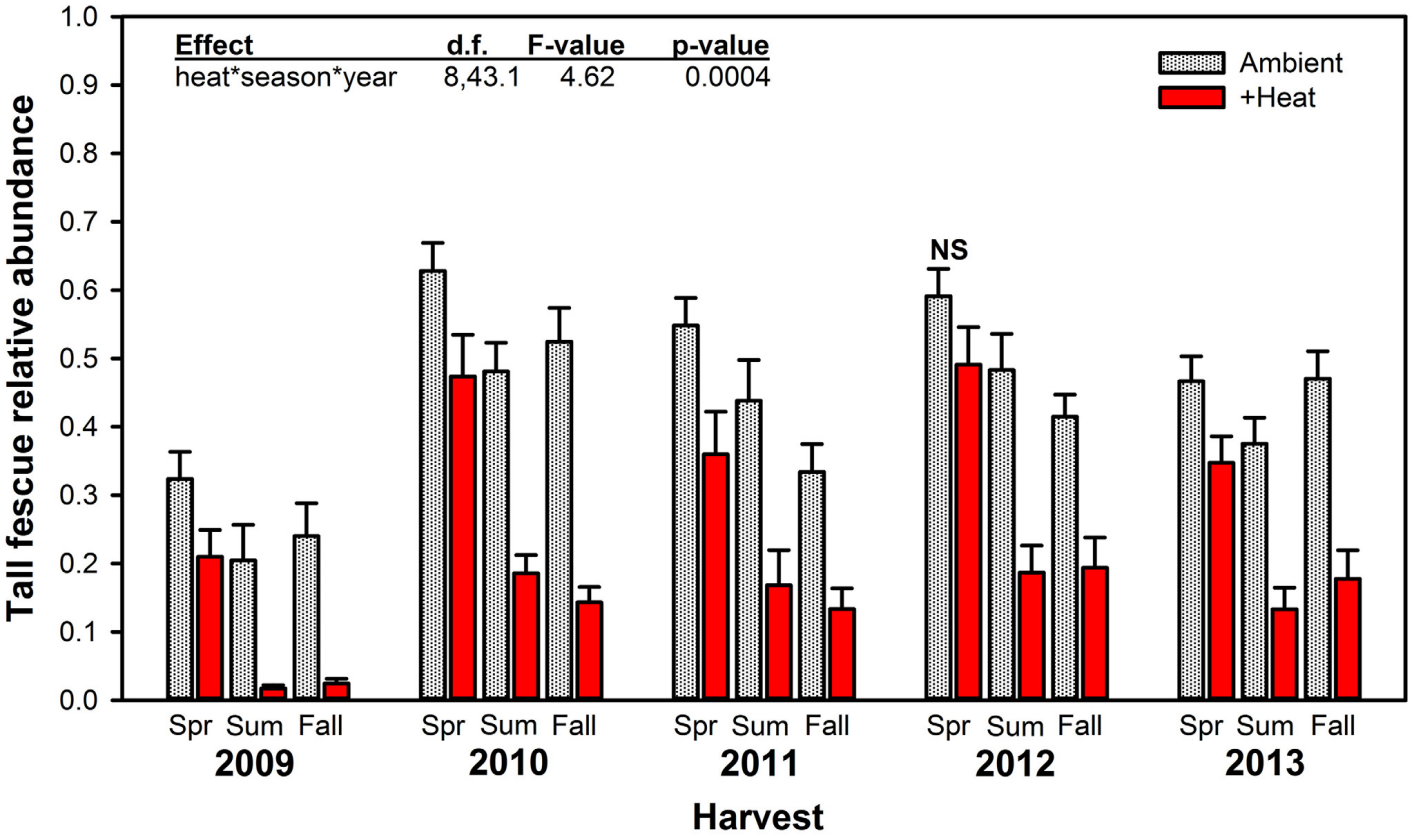
**Average (±SE) relative abundance of tall fescue (g tall fescue biomass/g total biomass) measured in heated (+3°C) vs. non-heated, ambient temperature treatments at every harvest period (Spring, Summer, and Fall) for all 5 years of the study**. The highest order significant interaction among the main effects of heat, season, and year is provided (see Table 2 for full statistical results). NS indicates the only harvest period where significant effects of heat were not observed.

Warming significantly reduced tall fescue relative abundance across the entirety of the project, except for the Spring 2012 harvest period when there was no significant difference between +heat and non-heated treatments (Figure 2). Even in June (Spring) 2009, after only a month of +heat treatments, tall fescue relative abundance was depressed by ~35% compared to non-heated plots. This immediate treatment effect did not reflect pre-existing differences in tall fescue abundance across plots, as visual cover estimates made in October 2008 indicated treatments were similar: 23.6% tall fescue ± 3.8—control; 26.3% ± 9.0—+heat; 27.6% ± 4.3—+precip; 24.0% ± 4.3—+heat+precip; and 25.6% vs. 25.2% for ambient temperature treatments vs. heated (average ± SE). Tall fescue relative abundance increased for both +heat and non-heated plots after the first year (by 217 and 132%, respectively) and remained relatively stable across years thereafter (averaging 36.4% across treatments). Tall fescue was most abundant in the Spring harvests for all treatments, and the difference between ambient temperature and +heat plots increased as the growing season progressed from Spring to the Summer and Fall harvests across all years of the study. Surprisingly, despite the +precip treatment increasing soil moisture by 8.5, 21.7, and 12.9% over control, +heat, and +heat+precip, respectively, over the course of the study, tall fescue relative abundance was not significantly affected by the addition of precipitation alone (*p* = 0.1029; Table 2). Although added precipitation increased soil moisture in the +heat+precip treatment over that of the +heat alone (by 11.2% over the course of the study), especially in summer, only a marginally significant interactive treatment effect (*p* = 0.0546 for heat × precip) on tall fescue abundance was identified (Table 2).

**Table 2:** **Statistical results of the effects of the climate treatments (+heat and +precip), season, year, and their interactions on tall fescue relative abundance in a mixed species pasture**.

**Effect**	**Num,Den *df***	***F*-value**	**Pr > *F***
Heat	1,33.7	51.68	**<0.0001**
Precip	1,33.7	2.81	0.1029
Heat^*^Precip	1,33.7	3.96	0.0546
Season	2,24.4	75.29	**<0.0001**
Heat^*^Season	2,24.4	9.33	**0.0010**
Precip^*^Season	2,24.4	1.89	0.1729
Heat^*^Precip^*^Season	2,24.4	1.16	0.3317
Year	4,26.6	48.10	**<0.0001**
Heat^*^Year	4,26.6	1.87	0.1449
Precip^*^Year	4,26.6	1.49	0.2344
Heat^*^Precip^*^Year	4,26.6	0.21	0.9283
Season^*^Year	8,43.1	12.51	**<0.0001**
Heat^*^Season^*^Year	8,43.1	4.62	**0.0004**
Precip^*^Season^*^Year	8,43.1	1.70	0.1269
Heat^*^Precip^*^Season^*^Year	8,43.1	1.41	0.2197

*Significant main effects and interactions are bolded*.

**Figure 3:**
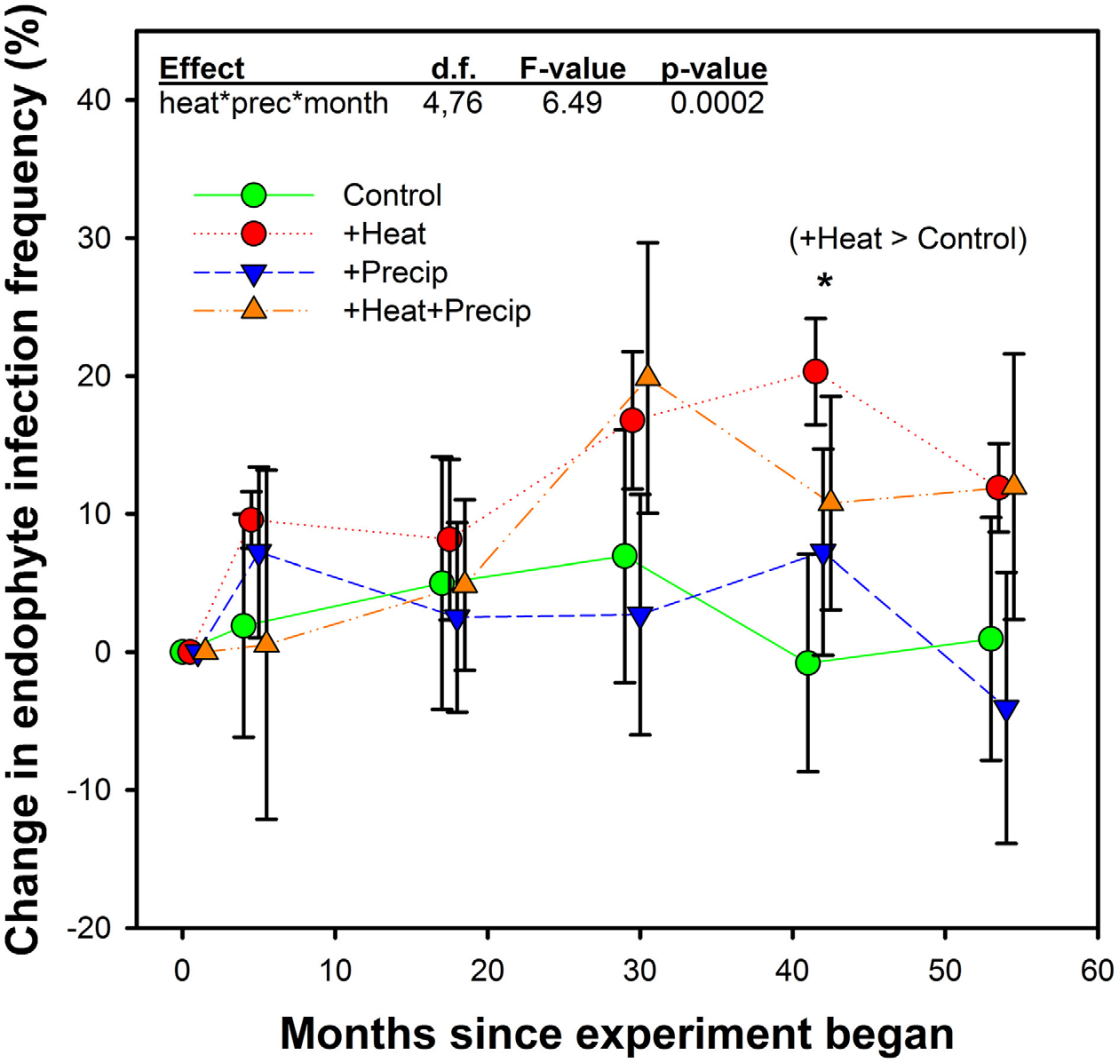
**Average (±SE) change in endophyte infection frequency within the tall fescue population in each plot over the 5 years study in heated and non-heated, ambient temperature treatments**. Points are slightly offset to clarify presentation, but measurements occurred at the same time for all treatments. Initial endophyte infection frequency averaged 41.1% (±3.3) across the site. Plot-level initial (June 2009) individual values were set as the zero point in this figure, and change from these values were calculated for measurements which occurred in Octobers from 2009–2013. The highest order significant interaction between heat, precipitation, and month since project began is shown, along with the only significant treatment comparison. See Table 2 for full statistical results. ^*^p = 0.0346.

In contrast to our hypothesis that +heat treatments would favor the survival and dominance of E+ individuals over E− within the tall fescue population, we found relatively limited effects of warming on changes in EIF over time (Table 3, Figure 3).

**Table 3:** **Statistical results of the effects of the climate treatments (+heat and +precip), month since the project began, and their interactions on the change in tall fescue endophyte infection frequency from the initial harvest June 1, 2009**.

**Effect**	**Num,Den *df***	***F*-value**	**Pr > *F***
Heat	1,76	1.49	0.2260
Precip	1,76	0.06	0.8073
Heat^*^Precip	1,76	0.09	0.7713
Month	4,76	3.13	**0.0194**
Heat^*^Month	4,76	2.31	0.0651
Precip^*^Month	4,76	0.09	0.9844
Heat^*^Precip^*^Month	4,76	6.49	**0.0002**

*Bolding indicates significant main effects and interactions*.

While a significant heat × precip × month since experiment began interaction was found, comparison tests identified only one significant mean separation. In October 2012, tall fescue within the +heat plots tested 20% more endophyte infected than in the beginning of the project, while the control plot EIF appeared to have not changed. However, this apparent warming effect disappeared the next year, as there were no significant treatment differences identified in October 2013 samples. Differences in EIF across time within a temperature treatment did occur (e.g., for +heat, changes in EIF in October 2012 were greater than in October 2010; for +precip, changes in EIF was greater in October 2012 than in October 2013; and for +heat+precip plots, changes in EIF was greater in October 2011 than in October 2009, 2010, and 2012).

### Alkaloids

**Figure 4:**
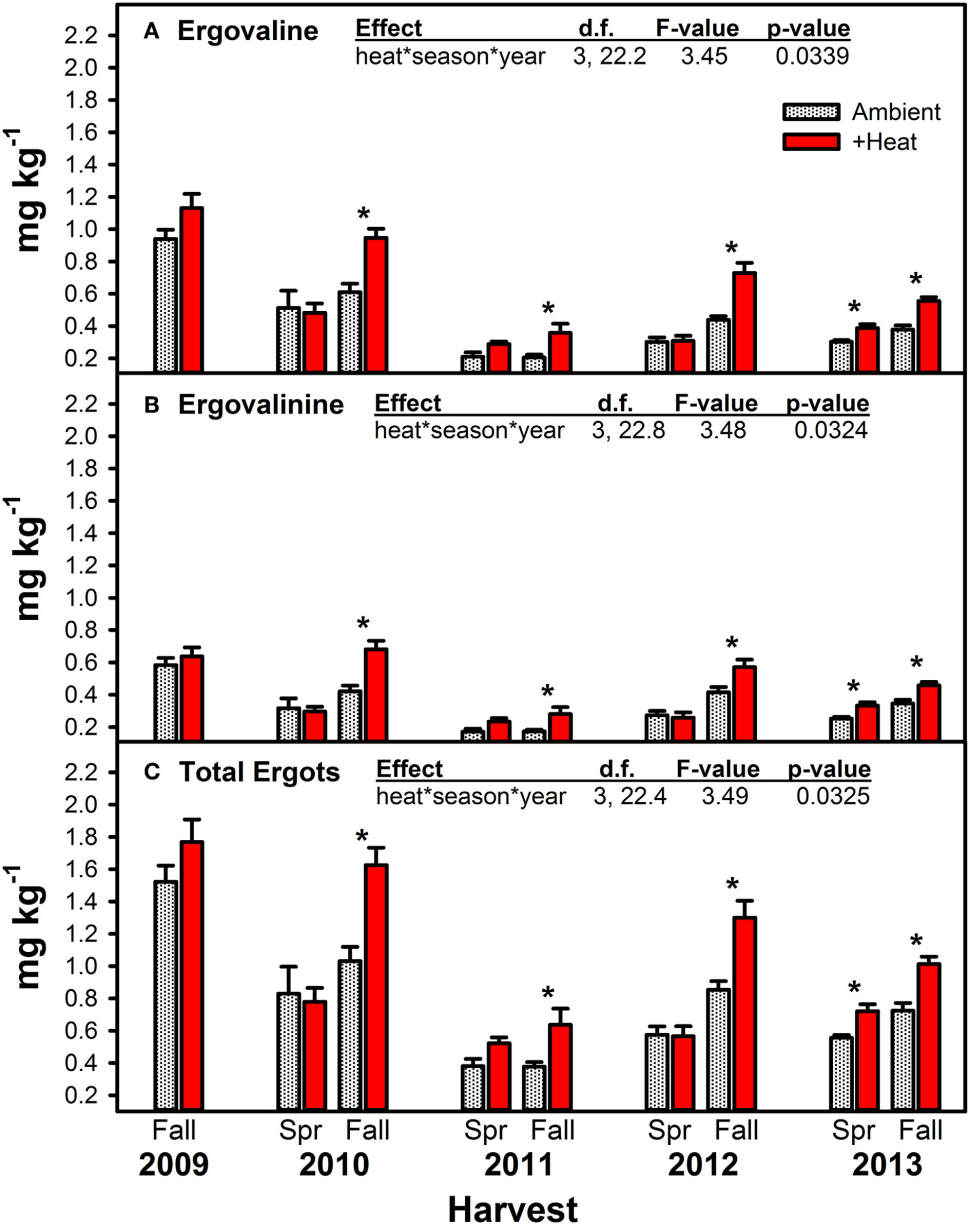
**Average (±SE) concentrations of ergovaline (A), ergovalinine (B), and the sum of these two ergot alkaloids (C) measured in endophyte-infected tall fescue tillers harvested from heated and non-heated, ambient temperature treatments in Spring and Fall of all years of the study**. Spring 2009 is missing due to lab error. The highest order significant interaction between heat, season, and year is shown (see Table 4 for full statistical results). Harvest periods with significant differences between treatments are indicated with an “^*^.”

Warming significantly increased concentrations of both measured ergot alkaloids and their sum, but this effect was primarily limited to the Fall harvested material (Figure 4, Table 4). Except for Spring 2013, the last year of the study, no warming effect was observed in Spring harvested material. The Spring 2013 trend was similar to Fall effects: +heat treatments had higher concentrations of ergot alkaloids than the non-heated, ambient temperature treatments. The magnitude of the warming induced increase in ergot alkaloids varied over time from +20.4 to +74.1% for ergovaline and +31.6 to +61.8% for ergovalinine. For both compounds, warming effects were strongest in Fall 2010, 2011, and 2012.

**Table 4:** **Statistical results of the effects of the climate treatments (+heat and +precip), season, year, and their interactions on the concentrations of ergovaline, ergovalinine, and their sum (total ergots measured in this study) in endophyte infected tall fescue tillers**.

**Effect**	**Ergovaline**			**Ergovalinine**			**Total ergots**		
	**Num,Den**			**Num,Den**			**Num,Den**		
	***df***	***F*-value**	**Pr > *F***	***df***	***F*-value**	**Pr > *F***	***df***	***F*-value**	**Pr > *F***
Heat	1,17.2	20.9	**0.0003**	1,26.2	13.66	**0.0010**	1,20.5	18.67	**0.0003**
Precip	1,17.2	0.84	0.3727	1,26.2	0.40	0.5302	1,20.5	0.68	0.4207
Heat^*^Precip	1,17.2	1.60	0.2226	1,26.2	1.07	0.3095	1,20.5	1.45	0.2427
Season	1,20.4	57.14	**<0.0001**	1,20.0	67.68	**<0.0001**	1,20.2	65.08	**<0.0001**
Heat^*^Season	1,20.4	18.89	**0.0003**	1,20.0	12.94	**0.0018**	1,20.2	17.07	**0.0005**
Precip^*^Season	1,20.4	0.00	0.9807	1,20.0	0.08	0.7788	1,20.2	0.02	0.8911
Heat^*^Precip^*^Season	1,20.4	0.01	0.9062	1,20.0	0.03	0.8720	1,20.2	0	0.9940
Year	4,17.4	35.83	**<0.0001**	4,18.1	22.3	**<0.0001**	4,17.5	30.65	**<0.0001**
Heat^*^Year	4,17.4	0.21	0.9319	4,18.1	0.80	0.5382	4,17.5	0.32	0.8638
Precip^*^Year	4,17.4	0.65	0.6368	4,18.1	0.42	0.7937	4,17.5	0.56	0.6951
Heat^*^Precip^*^Year	4,17.4	1.37	0.2858	4,18.1	0.78	0.5532	4,17.5	1.12	0.3801
Season^*^Year	3,22.2	10.24	**0.0002**	3,22.8	11.36	**<0.0001**	3,22.4	10.93	**0.0001**
Heat^*^Season^*^Year	3,22.2	3.45	**0.0339**	3,22.8	3.48	**0.0324**	3,22.4	3.49	**0.0325**
Precip^*^Season^*^Year	3,22.2	0.56	0.6465	3,22.8	0.80	0.5087	3,22.4	0.59	0.6228
Heat^*^Precip^*^Season^*^Year	3,22.2	0.64	0.5951	3,22.8	0.05	0.9843	3,22.4	0.29	0.8338

*Bolding indicates significant main effects and interactions*.

**Figure 5:**
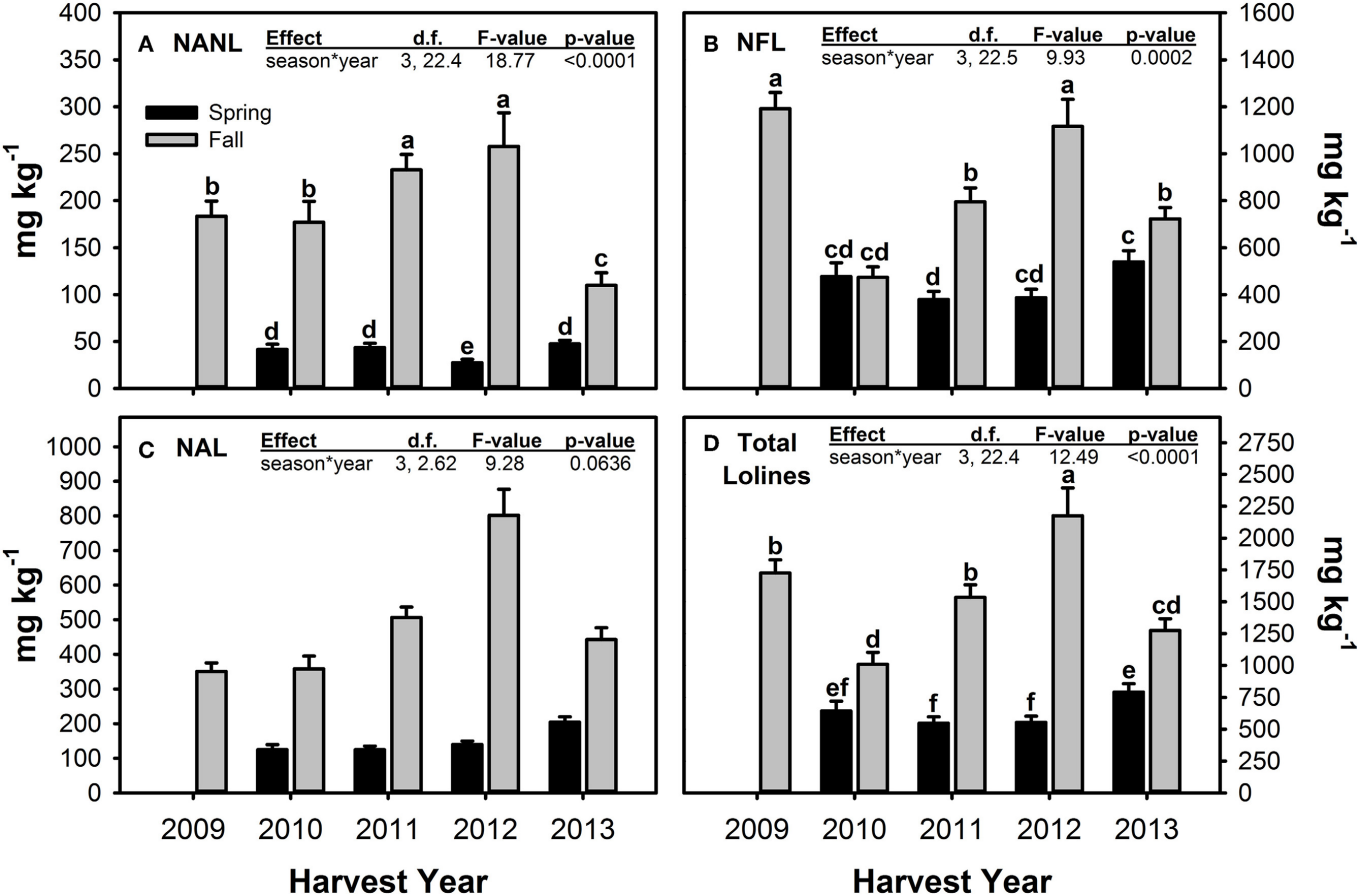
**Concentrations of *N*-acetyl norloline—NANL (A), *N*-formyl loline—NFL (B), *N*-acetyl loline—NAL (C), and the sum of these three loline alkaloids (D) measured in endophyte-infected tall fescue tillers harvested in Spring and Fall of all years of the study and averaged (±SE) across all climate treatments**. Spring 2009 is missing due to lab error. For loline alkaloids with a significant season × year interaction term, different letters indicate significant differences across all seasons and years of the study.

For the measured loline alkaloids, Fall harvested material tended to have significantly greater concentrations than Spring material (Figure 5, Table 5). The magnitude of difference between the seasons varied across years (hence the significant season × year interactions) and for the different compounds measured. NFL was the only loline alkaloid that had similar concentrations in Spring and Fall harvested material, and this only occurred in one year—2010. For all three loline compounds, Fall 2012 had the highest measured concentrations.

**Table 5:** **Statistical results of the effects of the climate treatments (+heat and +precip), season, year, and their interactions on concentrations of *N*-acetyl norloline (NANL), *N*-formyl loline (NFL), *N*-acetyl loline (NAL), and their sum (total lolines measured in this study) in endophyte infected tall fescue tillers**.

**Effect**	**NANL**			**NFL**			**NAL**			**Total lolines**		
	***df***	***F*-value**	**Pr > *F***	***df***	***F*-value**	**Pr > *F***	***df***	***F*-value**	**Pr > *F***	***df***	***F*-value**	**Pr > *F***
Heat	1,21.5	18.51	**0.0003**	1,17.3	6.94	**0.0173**	1,8.75	16.85	**0.0028**	1,20.9	16.01	**0.0007**
Prec	1,21.5	1.54	0.2274	1,17.3	3.49	0.0788	1,8.75	0.28	0.6109	1,20.9	2.74	0.1127
Heat^*^Prec	1,21.5	2.77	0.1107	1,17.3	1.53	0.2322	1,8.75	5.55	**0.0436**	1,20.9	3.91	0.0614
Seas	1,22.7	175.47	**<0.0001**	1,22.6	90.85	**<0.0001**	1,2.13	397.27	**0.0018**	1,22.9	223.34	**<0.0001**
Heat^*^Seas	1,22.7	11.58	**0.0025**	1,22.6	3.64	0.0691	1,2.13	12.90	0.0632	1,22.9	9.07	**0.0062**
Prec^*^Seas	1,22.7	2.34	0.1403	1,22.6	10.45	**0.0037**	1,2.13	4.84	0.1510	1,22.9	8.84	**0.0068**
Heat^*^Prec^*^Seas	1,22.7	6.40	**0.0188**	1,22.6	16.78	**0.0005**	1,2.13	19.42	**0.0425**	1,22.9	19.53	**0.0002**
Yr	4,17.5	8.94	**0.0004**	4,17.6	14.71	**<0.0001**	4,3.34	8.18	**0.0474**	4,16.9	7.07	**0.0016**
Heat^*^Yr	4,17.5	0.52	0.7228	4,17.6	0.33	0.8546	4,3.34	0.90	0.5506	4,16.9	0.46	0.7637
Prec^*^Yr	4,17.5	1.20	0.3457	4,17.6	0.64	0.6410	4,3.34	0.69	0.6438	4,16.9	0.61	0.6615
Heat^*^Prec^*^Yr	4,17.5	1.11	0.3850	4,17.6	1.16	0.3610	4,3.34	1.54	0.3629	4,16.9	1.56	0.2312
Seas^*^Yr	3,22.4	18.77	**<0.0001**	3,22.5	9.93	**0.0002**	3,2.62	9.28	**0.0636**	3,22.4	12.49	**<0.0001**
Heat^*^Seas^*^Yr	3,22.4	1.39	0.2708	3,22.5	1.74	0.1875	3,2.62	2.21	0.2843	3,22.4	1.97	0.1472
Prec^*^Seas^*^Yr	3,22.4	1.36	0.2806	3,22.5	0.07	0.9747	3,2.62	0.08	0.9635	3,22.4	0.11	0.9550
Heat^*^Prec^*^Seas^*^Yr	3,22.4	1.19	0.3369	3,22.5	0.10	0.9596	3,2.62	0.38	0.7769	3,22.4	0.22	0.8798

*Bolding indicates significant main effects and interactions*.

**Figure 6:**
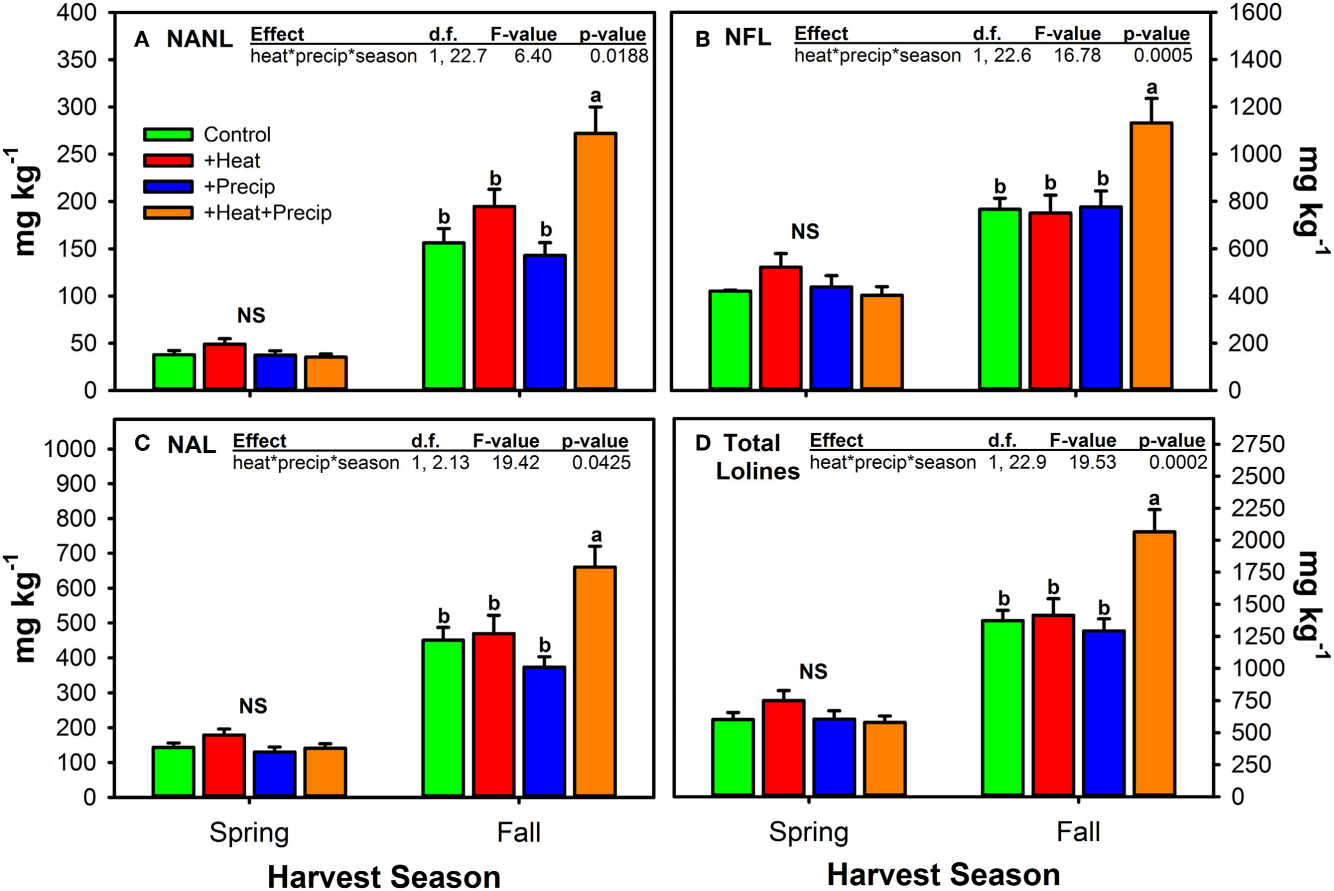
**Concentrations of *N*-acetyl norloline—NANL (A), *N*-formyl loline—NFL (B), *N*-acetyl loline—NAL (C), and the sum of these three loline alkaloids (D) measured in endophyte-infected tall fescue tillers from each of the four climate treatments averaged (±SE) by season of harvest (Spring or Fall) and across years of the study**. The highest order significant interaction between heat, precip, and season is shown (see Table 5 for full statistical results). NS indicates seasons when there were no statistically significant differences between the climate treatments. Otherwise, different letters illustrate significant differences between climate treatments.

Fall harvested material was also the only material that exhibited significant climate treatment effects on loline concentrations (Figure 6, Table 5). Across all 5 years of the study, in Fall harvested material, the combination of +heat and +precip produced significantly higher concentrations of all three measured loline compounds and their sum. None of the other three climate treatments (+heat alone, +precip alone, or the ambient control) differed from each other.

## Discussion

Our hypothesis that warming would reduce the relative abundance of tall fescue in this mixed species pasture, in part by increasing summer water limitation, a condition which is known to be stressful for this cool-season, C_3_ physiology grass (Belesky and West, 2009), was supported. Warming did reduce soil moisture, particularly in the summer and fall, and in every year of the study, warmed plots had significantly less tall fescue than ambient temperature plots. However, water limitation is only one potential factor explaining the warming associated decline in tall fescue abundance: competition from other plant species was most likely also important. Indeed, the increase in tall fescue abundance that was observed at the start of the second growing season (2010) across all treatments was associated with a dramatic decline in red clover, which was the dominant species in all treatments at the start of the project (data not shown). Additionally, in every year of the project, as seasonal temperatures increased from spring to summer and fall, warm-season, C_4_ physiology grasses (initially crabgrass, *Digitaria* spp., which recruited naturally from the seed bank, and later on in the project, Bermuda, which was planted) increased in dominance. These species were most abundant in the warmed treatments (data not shown) and were most likely strong competitors with tall fescue. Given the stressful conditions (both abiotic and biotic) that warming created for tall fescue, it is remarkable that it persisted in these plots for the duration of the study, at relatively stable levels.

One hypothesis that might explain how tall fescue managed to persist and effectively compete in the stressful warmed conditions of our project is that competitively superior, more environmental stress tolerant, E+ individuals were being selected for over time in these treatments, as E− individuals perished. However, this is not what we observed. Only at one point in time in our study, October 2012, after 4 years of experimental conditions, did we identify a significant treatment effect on the change in EIF over time. This effect was in the direction of what we hypothesized (the +heat treatment had 20% more E+ individuals than at the start of the experiment, whereas the EIF in the control treatment had not changed), but the significance of the effect disappeared the following year. In general, changes in EIF over time tended to be positive for +heat and +heat+precip treatments but were relatively small in magnitude (averaging 11.5% increase over time), while +precip and control treatments averaged almost no change (3.0% increase). The lack of a substantial change in EIF over time observed in this study, despite warming increasing abiotic and biotic stress, concurs with the results of Brosi et al. (2011b), who found that neither warming (+3°C) nor water limitation caused significant change in tall fescue EIF in a constructed, multi-species old field in Tennessee.

These results suggest that the importance of endophyte infection to tall fescue's ability to withstand and recover from elevated temperatures and water-limiting conditions may be less than prior work suggests. It is possible that our experimental treatments were not harsh enough to evoke the symbiotic benefits; however, both 2010 and 2012 were drier than normal years, with 2012 having one of the driest early growing seasons on record at the site (Mallya et al., 2013). Therefore, it is difficult to believe that the +heat treatment would not have produced harsh conditions for tall fescue at least at that time. These environmental conditions may have played into the Fall of 2012 being the only time period when significant treatment differences in the change in EIF over time were identified. It is also possible that endophyte benefits are conferred under more intense, but shorter duration, abiotic “extreme” events—rather than the long, continuous nature of our experimental climate manipulations. Additional work could further explore these possibilities.

Our results of relatively limited importance of endophyte infection to tall fescue abiotic stress tolerance are not unheard of: not all studies have shown benefits of endophyte infection to tall fescue under stressful conditions (MacLean et al., 1993; Richardson et al., 1993; Elbersen and West, 1996; Buck et al., 1997). Some have shown no effects (Arachevaleta et al., 1989; Belesky et al., 1989; Hill et al., 1996), and work performed in this same project found that E+ tall fescue experienced more mortality than E− after 1 month of +heat treatments (Brosi, 2011a) and that tall fescue's response to the treatments is under plant genetic control, in addition to being impacted by *Epichloë* presence and strain type (Bourguignon, 2013). We did not control for plant genetic background in this experiment, as we were interested in measuring changes to the population, and it is possible that differing fescue genotypes in our project responded to both climate treatments and endophyte presence in contrasting ways (West, 2007), which might have hampered our ability to detect an endophyte-associated response to the climate treatments. Complicated symbiotic responses to abiotic perturbations and their resulting effects on various ecological parameters are consistent with the growing body of knowledge regarding controls of grass—*Epichloë* interactions (Faeth and Saikkonen, 2007; West, 2007; Rudgers et al., 2010; Yurkonis et al., 2014), and will need to be factored into predictions regarding population-level grass symbiotic responses to climate change.

It is possible that the primary importance of the fungal endophyte symbiosis for tall fescue under altered climatic conditions will be conferred through interactions with herbivory. Field manipulations of herbivory have clearly demonstrated that endophyte presence confers an advantage under high herbivore pressure, such that significant increases in EIF of the tall fescue community were observed over time (Clay et al., 2005). In our project, in E+ individuals, we measured significant increases in both ergot and loline alkaloids in fall plant material from treatments representative of predicted future climates for the region. Because these compounds are toxic to herbivores, these results suggest that those herbivores capable of selecting for E− fescue or utilizing other plant species will do so, potentially altering plant competitive dynamics.

While assessing the response of herbivores to the climate change treatments was not the primary focus of our study, concurrent projects that monitored aphids, slugs, and small mammal seed predation and population dynamics, at varying times throughout the 5 years project, found few changes in herbivore numbers or feeding patterns related to the biotic and abiotic alterations that the climate change treatments produced. Rua et al. (2014) monitored aphids in May 2010 and found that there were more aphids in the +heat plots than the other treatments, but was unable to test for differences in aphid numbers between E+ and E− tall fescue due to low numbers of aphids being found on this material—a result similar to that of Brosi (2011a). Three years of slug manipulations and trapping at the site indicated that slug herbivory levels were low and that climate treatment effects on these herbivores were minor (Weber, 2014). Small mammal, primarily vole (*Microtus pennsylvanicus, M. ochrogaster*), population dynamics varied from year-to-year, as is common for these organisms (Chitty, 1960; Krebs and Myers, 1974), but seed predation was generally similar across plots (A.E. Carlisle, unpublished data). It is possible that the mowing regime or other environmental or site factors created conditions that minimized herbivory levels at our site, and may be one reason why changes in EIF were not observed, despite climate treatments increasing alkaloid concentrations and significantly altering the abiotic environment and plant communities. Changes in tall fescue EIF may have been more dramatic had selective, heavy grazing, rather than non-selective mowing, been practiced on site, though it should be noted that an experiment in southern Illinois found that mowing increased water limitation, which the authors hypothesized selected for higher EIF of the tall fescue in mowed compared to adjacent not mowed plots (Spyreas et al., 2001). The factors causing the observed lack of change in EIF in this experiment remain unknown.

Our project failed to incorporate increases in atmospheric CO_2_ concentrations, due to financial and infrastructure limitations, which is unfortunate as this factor will be an important component of future environmental conditions and may modify effects of warming and altered precipitation on tall fescue (Yu et al., 2012), and has been shown to be important in governing the fescue-endophyte symbiosis. Brosi et al. (2011b) found that elevated CO_2_ increased the occurrence of E+ tall fescue individuals in a mixed species stand but reduced the concentrations of both ergot and loline alkaloids. Ryan et al. (2014) also reported reduced alkaloid concentrations in E+ tall fescue in response to elevated CO_2_, despite measuring increased fungal DNA concentrations in these same individuals. In contrast to the Brosi et al. (2011b) work, we measured significant increases in ergot alkaloids in response to warming (Brosi measured no response of ergots to warming), and we found that lolines increased in concentration only when warming was accompanied by additional precipitation (Brosi found warming alone stimulated lolines, that dry, not wet conditions, increased loline concentrations, and there was no interaction between warming and water availability in that study). Differences in site conditions or experimental designs and treatments may account for these discrepancies, as nitrogen availability has been shown to alter the response of alkaloid concentrations to elevated CO_2_ in perennial ryegrass when infected with *E. festucae* var. *lolii* (Hunt et al., 2005).

Clearly, the *Epichloë*—tall fescue symbiosis will be affected by and respond to climate change factors. Given that all research performed to date on the subject has shown alkaloids to be responsive to the effects of elevated atmospheric CO_2_, warming, and drought/additional precipitation, and these biological compounds play key roles in governing herbivory and animal production in pastures, it is imperative that future work attempts to better characterize the interactive effects of these various climate change factors on alkaloid concentrations. Brosi et al. (2011b) is the only paper to date able to assess the interactive effects, and they found no significant interactions on any of the measured compounds, suggesting that the individual climate treatment main effects cancel each other out. However, replication was limited in the Brosi work (*n* = 3), and the above-mentioned differences between our results and theirs for warming and precipitation manipulations suggest that additional factors may be governing tall fescue—endophyte alkaloid responses. If elevated atmospheric CO_2_ does not mitigate the effects of warming and changes in precipitation on alkaloid concentrations in E+ tall fescue, our data suggest that those herbivores that are incapable of altering forage selection (e.g., cattle grazing highly infected tall fescue dominated pastures) will likely consume higher levels of these toxic compounds, and they will do so at an already hot time of the year in many places (late Summer/Fall), when there are significant challenges to maintaining ideal thermal conditions for optimal animal functioning (Spiers et al., 2005). Additional research evaluating pasture management options to reduce consumption of toxic alkaloids (e.g., the use of novel, non-toxic endophyte infected material, specific grazing strategies, and/or other forage species, especially warm season grasses; Aiken and Strickland, 2013) while sustaining pasture-based animal production under future climatic conditions seems warranted.

## Author contributions

Rebecca L. McCulley and Jim A. Nelson conceived the research idea and designed the experiment. All authors were involved in aspects of data acquisition, analysis, and interpretation. All authors contributed to writing the manuscript, approved the final version, and are accountable for the data presented and interpretation therein.

### Conflict of interest statement

The authors declare that the research was conducted in the absence of any commercial or financial relationships that could be construed as a potential conflict of interest.
